# Experimental pain and fatigue induced by excessive chewing

**DOI:** 10.1186/s12903-020-01161-z

**Published:** 2020-06-29

**Authors:** Samaa Al Sayegh, Ioanna Vasilatou, Abhishek Kumar, Ceva Al Barwari, Lars Fredriksson, Anastasios Grigoriadis, Nikolaos Christidis

**Affiliations:** 1 Division of Oral Diagnostics and Rehabilitation, Department of Dental Medicine, Karolinska Institutet, Box 4046, SE-141 04 Huddinge, Sweden; 2 Scandinavian Center for Orofacial Neurosciences (SCON), Huddinge, Sweden; 3 Department of Clinical Oral Physiology at the Eastman Institute, Folktandvården Stockholms län AB, SE-113 24 Stockholm, Sweden

**Keywords:** Pain model, Fatigue, Chewing gum, Sex differences, Temporomandibular disorders

## Abstract

**Background:**

The study was aiming to optimize excessive gum chewing as an experimental model to induce jaw muscle pain and fatigue similar to those in painful TMDs with durations that would allow immediate investigations of jaw-motor function. Further, if any sex differences would be detected in the expression of pain.

**Methods:**

This randomized, double blinded study included 31 healthy participants of both sexes. A standardized chewing protocol of either 40- or 60-min of chewing was used with a wash-out period of 1 week. Subjective fatigue, pain characteristics and functional measures were assessed. For statistical analyses, Wilcoxon Signed Rank test, Mann–Whitney Rank Sum test and Friedman’s ANOVA with Tukey post-hoc test were used.

**Results:**

High subjective fatigue scores that lasted up to 20 min after the end of the trial were significantly induced both in the 40- and 60-min chewing trials (P <  0.001*). Significant but mild pain was induced only in the 60-min trial (P = 0.004*) and only in men (P = 0.04*). Also, the induced pain area was significantly bigger in the 60-min trial (P = 0.009*). However, this increase in pain and pain area did not last to the first 10-min follow-up. There were no significant differences neither between the 40- and 60-min chewing trials, except regarding the pain area (P = 0.008*), nor between the sexes.

**Conclusion:**

Taken together, excessive chewing in its current form does not seem to be a proper pain experimental model. The model needs further adjustments in order to mimic TMD-pain especially in women and to prolong the pain duration.

## Background

The methods to assess pain and its treatment approaches that currently are available are sub-optimal due to limited understanding of the aetiology and pathophysiology of chronic pain [1]. It is also unclear how pain from the temporomandibular region affects jaw-motor function and oral fine-motor performance. Limited jaw function is a common complaint among adults, where the prevalence of temporomandibular disorders (TMD) reaches levels of 10–20% [2–5]. Further, TMD is considered a common source for chronic musculoskeletal pain with higher prevalence among women than in men [4, 5]. Further, patients with TMD pain of muscular origin usually report their pain as an exercise-alike pain, which also often is accompanied with a component of fatigue or exertion [6, 7]. A standardized experimental setting with a homogenous group, would improve our understanding about pain mechanisms especially jaw muscle pain, as well as how this jaw muscle pain affects jaw function. Such an experimental model would also decrease the risk of confounders when evaluating the possible outcomes as well as assessing the trustworthiness of the findings.

Various types of exogenous and endogenous experimental pain models are used to mimic clinical pain [8, 9]. However, several of these models have been shown to be partly inexpedient since they are not fully mimicking the chronic clinical pain condition [2, 9]. Pain and fatigue in the masticatory muscles [6, 7] are more similar to exercise-induced pain rather than pain evoked by exogenous techniques, which is more intense and short-lasting [2, 9–11]. Prolonged exercise that exceeds a muscle’s capacity will lead to muscle soreness and fatigue. Overloading of muscles beyond an already achieved fatigue without time for recovery will lead to traumatized muscle tissue. The disadvantage of the ischemic stimulation is that it also involves other muscles and/or surrounding tissues than the intended experimental area.

Previous studies indicate that excessive chewing results in increased muscle fatigue scores and pain, with the majority of the participants showing signs of myofascial pain or arthralgia [11, 12]. To our knowledge there are no studies using chewing gum as a pain-inducing model where different chewing durations and sex differences have been investigated. Therefore, the primary aim of the study was to optimize excessive gum chewing as an experimental model to induce jaw muscle pain and fatigue similar to those in painful TMDs with durations that would allow immediate investigations of jaw-motor function. Further, if any sex differences would be detected in the expression of pain. We hypothesized that excessive hard gum chewing would induce jaw muscle pain and fatigue mimicking clinical pain and subjective fatigue in TMD patients. Secondarily and thirdly, the induced pain and fatigue would last longer in women than in men, and therefore allow further investigations of the jaw-motor function in women. However, a longer chewing duration would be needed in order to induce fatigue and pain in men.

## Methods

This prospective, randomized, controlled, double blind trial (RCT) follows the consolidated standards of reporting trials (CONSORT) statement [13–15] and was conducted at the Department of Dental Medicine at Karolinska Institutet, Huddinge, Sweden during the period of September 2017 until November 2017 and consisted of two sessions with a wash-out period of 1 week.

### Participants

**Fig. 1:**
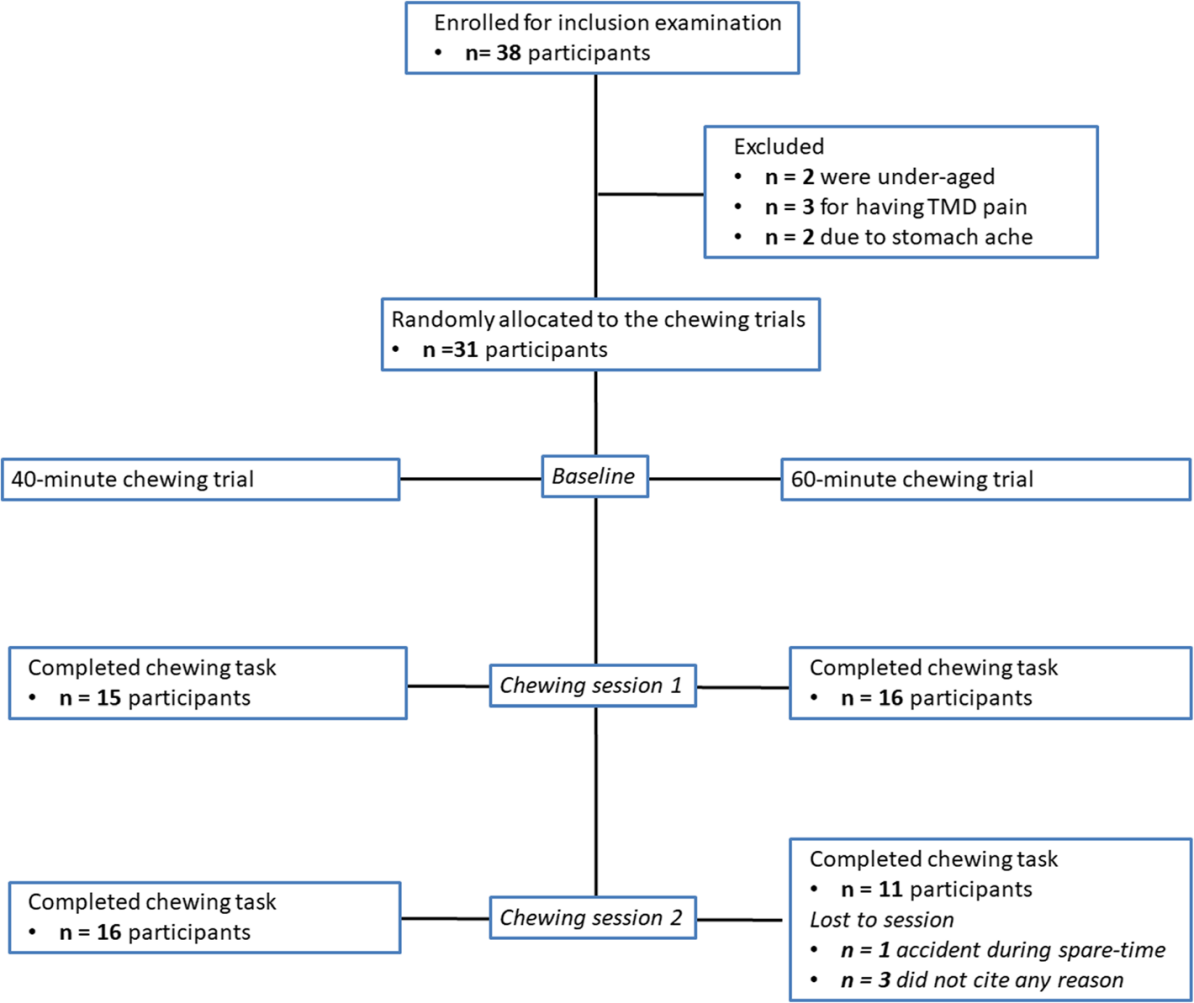
CONSORT flowchart. Flowchart of the 31 participants throughout the study

In total 38 persons were enrolled for inclusion examination and all were screened by one examinator (IV). Thirty-one healthy participants were found eligible and included in the study, 15 healthy men with a mean (SD) age of 27 (5.4) years and 16 healthy age-matched women aged 25 (4.3) years (Table 1), no one declined participation (Fig. 1). Twenty six participants were required according to a non-inferiority power calculation (https://www.sealedenvelope.com/power/binary-noninferior/) to achieve a significance level (α) of 0.05 and power (β) of 80% excluding a difference of more than 30% in pain intensity and duration in favor for the 60-min trial [11, 12].

The inclusion criteria were: a) age over 18 years; and b) good general health. The exclusion criteria were: 1) a diagnosis of myalgia, myofascial pain, arthralgia, headache attributed to TMD, degenerative joint disease, painful clicking or locking, all according to the Diagnostic Criteria for Temporomandibular Disorders (DC/TMD) [4]; 2) additional palpatory tenderness of the masseter, temporal muscles or over the temporomandibular joint (TMJ); 3) clinically visible dental pathology or mobility, tooth wear grade 3 = exposure of pulp or secondary dentine according to the simplified scoring criteria for tooth wear index I [16], malocclusion, edentulous areas or dentures; 4) general chronic pain conditions, systemic inflammatory diseases (i.e. rheumatoid arthritis, fibromyalgia, etc.), neuropathic pain or neurological disease; 5) whiplash associated disorder; 6) use of any medication that might influence the response of pain i.e. analgesics during 24 h preceding the trial, use of cannabinoids, or any medication that might influence the neurological function; 7) self-reported bruxism and chewing gum for more than 30 min on a daily basis, since these activities may affect the chewing muscles’ resistance to fatigue [17]; 8) allergy to any of the contents in the chewing gum; 9) pregnancy; and 10) cognitive or physical disability that prevent participation.

### Experimental protocol

Participants filled in questionnaires regarding psychosocial variables: anxiety (generalized anxiety disorder scale-7; GAD-7) [18], depression (the patient health questionnaire for depression-9; PHQ-9) [19], physical/somatic symptoms (the patient health questionnaire for physical symptoms-15; PHQ-15) [20], stress (perceived stress scale-10; PSS-10) [21] and pain catastrophizing (pain catastrophizing scale-13; PCS-13) [22]. Information regarding use of contraceptives and phase of the women’s menstrual cycle was obtained in order to take the hormonal variation into consideration [5]. All participants were clinically examined according to the standardized examination protocol of diagnostic criteria for temporomandibular disorders (DC/TMD-Axis I) prior to inclusion but also at the end of the chewing task as well as at the 1-h follow-up and the 2-h follow-up. Only one examiner (IV; trained in DC/TMD) performed all examinations, and she was blinded to the duration of the chewing trials. Counterbalancing was used to control for order effects. Therefore, participants were randomized, in blocks of four using a digital tool (www.randomization.com) and blinded to start either with a 40-min or a 60-min chewing trial and vice versa after a wash-out period of 1 week, by a researcher not participating in data collection (NCh). Hence, order effects would occur equally in both groups and balance each other out in the results. Based on pilot studies performed with chewing durations of 20, 40, 60 and 100 min (unpublished results) we chose to use duration of the chewing tasks limited to 40 versus 60 min. This choice was partly based on the outcome that there were no differences between the 60 and 100 min groups regarding any of the variables but there were unwished side-effects of intestinal load/discomfort after the 100 min trial. The 20-min group did not result in any induced pain or fatigue/exertion. Also, future clinical experiments should have a reasonable duration and consequently being performed the same day. Further, it is well-known that women are more susceptible to pain [5], a longer duration would result in a high number of drop-outs among them.

The participants chewed five new chewing gums divided in 5-min chewing bouts (ELMA® sugar free, Mastiha, Chios, Greece; 5 × 1,4 g = 7 g) [11, 12, 23, 24]. The participants were instructed to continuously chew without rest on their dominant habitual masticatory side, following their natural chewing pattern. The examiner IV monitored the chewing procedure during the entire task. In order to reduce the risk that a standardized rate could influence the results we chose to use the dominant habitual masticatory side and the participants’ natural chewing pattern. The gain of this method is that in daily life there are many individuals who are “fast-chewers” and if the chosen chewing rate in an experiment happens to be “slower” than those participants’ natural chewing rate then the results would be misleading since no subjective fatigue or pain would be detected [25].

**Fig. 2:**
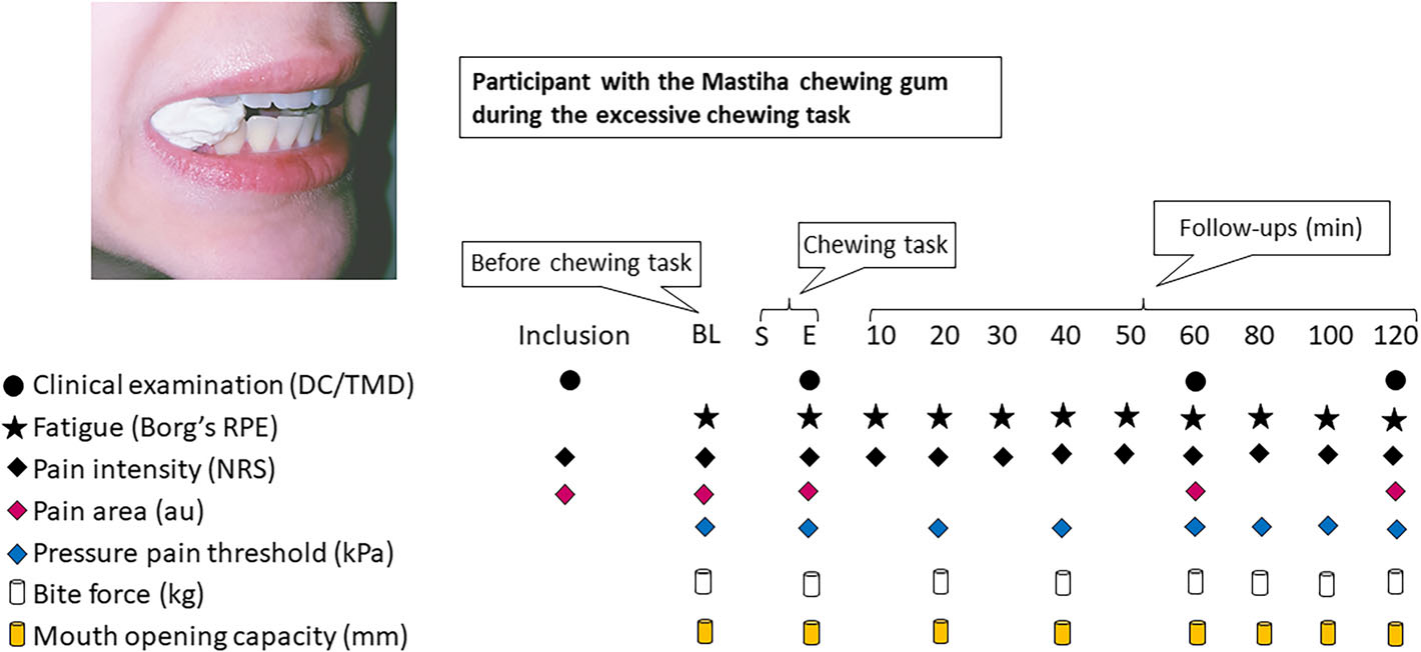
Flow-chart of the experimental protocol. This flow-chart illustrates the experimental protocol. BL = baseline; S = start of chewing task; E = end of chewing task; min = minuteDC/TMD = Diagnostic Criteria for Temporomandibular Disorders; Borg’s RPE = Rating of Perceived Exertion; NRS = Numeric Rating Scale; au = arbitrary units; kPa = kiloPascal; kg = kilogram; mm = millimeter

The values of jaw subjective fatigue (Borg’s Rating of Perceived Exertion; Borg’s RPE) and pain intensity (Numeric Rating Scale; NRS) were monitored and assessed at baseline, every 10 min during, immediately after and every 10 min after the chewing task during the 1-h follow-up and every 20 min during the 2-h follow-up. Pain drawings were assessed at baseline, at the end of the chewing task and after 1 and 2 h respectively following the chewing tasks. Further, at baseline, every 20 min during, immediately after as well as every 20 min after the chewing task up till 2 h of follow-up, pressure pain thresholds (PPT) over the masseter and temporal muscles as well as the index finger (reference point), maximum voluntary bite force (MVBF) and maximum voluntary mouth opening capacity (max MOC) were assessed. One examiner (IV) performed all assessments and was blinded to the duration of the chewing trials. The experimental protocol and time points of measurement variables are illustrated in Fig. 2.

### Assessment of jaw subjective fatigue, pain variables and pressure pain threshold

Subjective fatigue was assessed using Borg’s RPE [6–20], where 6 is extremely easy effort and 20 is maximum effort [26].

Pain intensity and peak pain were assessed using a NRS (0–10) where the end-points were 0 = no pain and 10 = worst imaginable pain [27].

A lateral chart of the face for both the right and left sides separately as well as intra-orally was used for assessing the pain spread. The participants were asked to mark all the areas in which they sensed pain on the chart by drawing a ring around the painful space. The drawings were later scanned and the Adobe Photoshop CC software (version 19.1.3, Adobe Systems Incorporated, San Jose, CA, USA) was used to count the pixels within the marked total area in arbitrary units (au).

An electronic pressure algometer (Somedic Sales Hörby AB, Sweden) was used over the masseter and temporal muscles bilaterally to assess pressure pain threshold (PPT). The algometer is supplied with a soft rubber tip with a surface of 1 cm^2^, which was applied perpendicular to the participants’ skin surface. The participants were asked to clench and relax in order to determine and mark the most prominent area of the masseter belly and the anterior temporal muscle which would be the site for the pressure application. The participants were also instructed to press a button immediately as the sensation of pressure turned into pain. The participants’ head was supported on the opposite side by the examiner’s hand. The pressure was increasing with a rate of 30 kPa/s [11, 28, 29]. The electronic pressure algometer was calibrated before each trial. PPT was assessed by one calibrated examiner (IV), and repeated twice over each muscle site at each assessment and the mean value was used for data analyses.

### Assessment of functional measures

In order to assess maximum voluntary bite force in Kilogram (Kg), a bite force transducer (41.0 × 12.0 × 5.0 mm, length × width × height, Aalborg University, Aalborg, Denmark) was used. The bite force transducer was covered with 1 mm rubber in order to avoid any cross contamination and reduce the risk of tooth fracture and inserted between the first or second molars either on the right or left side depending on each participant’s dominant habitual masticatory side.

The maximum voluntary mouth opening capacity, inclusive the vertical overbite, was assessed according to DC/TMD-Axis I in millimeters.

### Statistical analyses

The normality of all data was tested with the Shapiro-Wilk test. The data showed a non-normal distribution and a skewness to the right, all except age. Therefore, non-parametric tests were used to analyze data and all data except age are presented as median (interquartile range; IQR). The PPT, BF and MOC values were normalized and presented as the percentage change from baseline values. The data were analyzed with the SigmaStat software (version14.0; Systat Software Inc., San Jose, CA, USA) and for all tests, the level of significance was set at *P* <  0.05 for within groups comparisons and *P* <  0.005 for subjective fatigue and pain intensity, < 0.013 for pain area and <  0.006 for the rest of the variables for between groups comparisons after applying Bonferroni corrections. The data from the four participants that did not attend at the second session (all lost at the 60-min chewing trial) were analyzed for their first session, the 40-min chewing trial, and were handled as missing data for their second session, the 60-min chewing trial.

For baseline and between groups comparisons, Wilcoxon Signed Rank test was used to test differences between trials and Mann–Whitney Rank Sum test was used to test sex differences as well as testing differences between the sessions. For within groups comparisons, the nonparametric Friedman’s analysis of variance for repeated measures with Tukey post-hoc test for the associated multiple comparisons were used to test changes in all variables versus baseline. The factors included in the analyses were time (baseline, end of chewing task and follow-up time-points), trials (40-min chewing trial and 60-min chewing trial), sex (men and women) and sessions (session 1 and session 2).

## Results

### Participants

The psychosocial characteristics of all participants are presented in Table 1. All psychosocial variables were within a normal range, except for the physical/somatic symptoms and stress in women that were of mild grade. The physical/somatic symptoms were also significantly higher in women than men. The baseline values of subjective fatigue, pain characteristics and functional measures are presented in Table 2. Women displayed lower baseline PPT, BF and MOC than men. There were no significant differences between those starting with the 40-min or 60-min chewing trial (Tables 1 and 2).

**Table 1:** The table presents the age and psychosocial characteristics of the participants

	All		Men		Women		
Number of Participants	31		15		16		
Age	26 (4.90)		27 (5.51)		25 (4.19)		
	**All**	**40-min**	**60-min**	***P*-value**	**Men**	**Women**	**P-value**
GAD-7^a^	2.00 (4.00)	1.00 (3.00)	2.00 (3.00)	0.24	2.00 (4.00)	1.50 (4.75)	0.98
PHQ-9^b^	2.00 (5.00)	2.00 (4.00)	2.00 (5.50)	0.81	1.00 (5.00)	3.50 (3.00)	0.59
PHQ-15^c^	4.00 (7.00)	4.00 (8.00)	3.00 (7.75)	0.70	2.00 (5.00)	8.00 (7.50)	0.02*
PSS-10^d^	10.00 (13.00)	10.00 (17.00)	9.50 (12.25)	1.00	7.00 (10.00)	14.50 (9.75)	0.08
PCS-13^e^	2.00 (12.00)	2.00 (17.00)	2.00 (9.50)	0.94	2.00 (4.00)	5.50 (16.25)	0.42

Age expressed in mean (SD; standard deviation) and psychosocial variables in median (IQR; interquartile range) according to Axis II in Diagnostic Criteria of Temporomandibular Disorders. *P*-values refer to the comparisons between trials and sexes by Mann-Whitney Rank Sum test

* = significant difference P < 0.05

^a^GAD-7: Generalized Anxiety Disorder (7 Questions)

^b^PHQ-9: The Patient Health Questionnaire for Depression (9 Questions)

^c^PHQ-15: The Patient Health Questionnaire for Physical Symptoms (15 Questions)

^d^PSS-10: Perceived Stress Scale Scoring (10 Questions)

^e^PCS-13: Pain Catastrophizing Scale (13 Questions)

**Table 2:** The table presents the baseline values of fatigue, pain characteristics and functional measures

	All		P-value	40-min		P-value	60-min		P-value
	40-min	60-min		Men	Women		Men	Women	
**Fatigue**	6 (0)	6 (0)	0.88	6 (0)	6 (0)	0.15	6 (0)	6 (0)	0.12
**Pain Characteristics**									
Pain Intensity	0 (0)	0 (0)	1.00	0 (0)	0 (0)	1.00	0 (0)	0 (0)	1.00
Pain Area	0 (0)	0 (0)	0.63	0 (0)	0 (0)	0.45	0 (0)	0 (0)	0.20
Pressure Pain Thresholds									
PPT Masseter	165.50 (95.25)	183.75 (111.50)	0.16	214.50 (69.25)	140.38 (28.88)	0.02*	220.00 (116.25)	139.25 (81.06)	0.02*
PPT Temporal	218.75 (109.50)	215.25 (152.00)	0.34	297.50 (69.75)	208.63 (37.19)	0.01*	297.75 (118.50)	186.25 (41.00)	0.01*
PPT Reference	450.25 (184.00)	401.50 (197.30)	0.08	494.75 (316.00)	437.25 (182.69)	0.17	465.25 (331.00)	375.75 (138.44)	0.27
**Functional Measures**									
Max Bite Force	21.30 (14.50)	20.80 (25.30)	0.66	24.20 (32.10)	20.25 (5.88)	0.33	35.90 (26.80)	14.80 (16.18)	0.02*
Max Mouth Opening	55.00 (10.00)	55.00 (10.00)	0.36	60.00 (7.00)	52.00 (8.50)	0.003*	60.00 (6.00)	51.50 (10.50)	0.01*

The subjective fatigue was assessed in Borg’s RPE, the pain intensity in numeric rating scale (NRS), the pain area in arbitrary units (au), the pressure pain threshold in kilo Pascal (kPa), the maximum voluntary bite force in kilogram (kg) and the maximum voluntary mouth opening capacity in millimeters (mm). Data are expressed in median (IQR; interquartile range). P-values refer to the comparisons between trials by Wilcoxon Signed Rank and sexes by Mann-Whitney Rank Sum test. * = significant difference *P* < 0.005 for subjective fatigue and pain intensity, < 0.013 for pain area after applied Bonferroni Corrections and < 0.05 for the rest of the variables

### Subjective fatigue

**Fig. 3:**
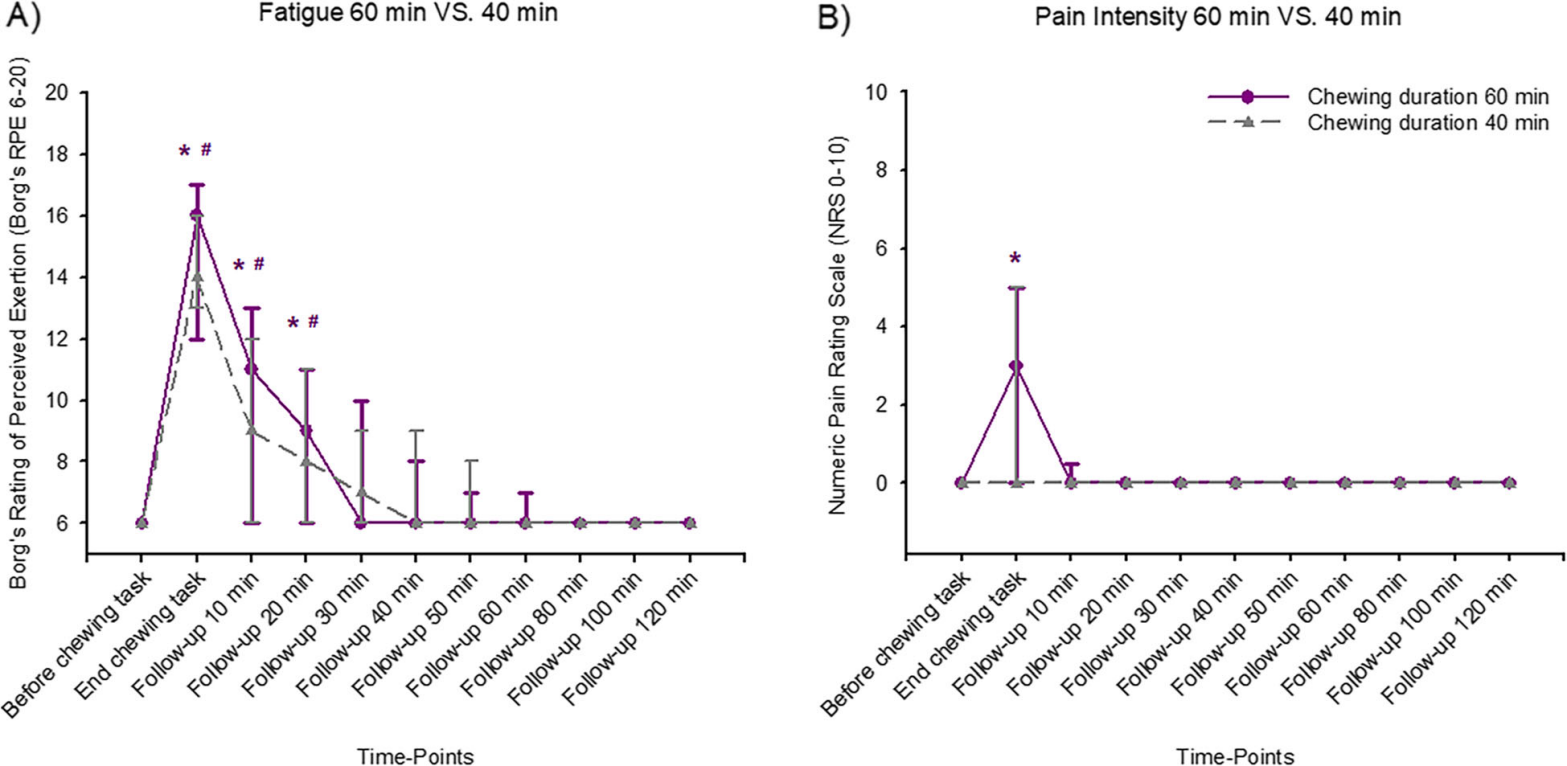
Changes in subjective fatigue and pain intensity by excessive gum chewing. In this figure, median (IQR) subjective fatigue (**a**) and pain intensity scores (**b**) are shown before, at the end of the chewing task and at follow-ups up to 120 min after.15 healthy men and 16 age-matched healthy women participated in 2 sessions of either 40 or 60 min of excessive chewing the Mastiha chewing gum. The subjective fatigue scores increased significantly after the chewing tasks. The chewing tasks during 40-min and 60-min induced significantly higher subjective fatigue scores than baseline. The significant increase lasted up till 20 min after the chewing tasks. *Significant difference compared to baseline for 60-min chewing task (Friedman ANOVA test/Tukey post-hoc; *P* <  0.05). #Significant difference compared to baseline for 40-min chewing task (Friedman ANOVA test/Tukey post-hoc; P <  0.05). The pain intensity increased significantly after the 60-min chewing task. At the end of the 60-min chewing task, significantly higher pain intensity scores than baseline was induced. The significant increase did not last up till 10 min after the chewing task. There was no significant increase in pain intensity after the 40-min chewing task. *Significant difference compared to baseline for 60-min chewing task (Friedman ANOVA test/Tukey post-hoc; *P* < 0.05)

Excessive chewing induced a significant increase in subjective fatigue that lasted for 20 min after completed chewing, in both the 40 and 60 min trials when compared to baseline (Table 3 and Fig. 3a). There were no significant difference in subjective fatigue when the two chewing trials were compared (Table 4).

**Table 3:** Changes compared to baseline in all measures in 40- and 60- min trials in all 31 participants

	40-min Trial		***P***-value	60-min Trial		***P***-value
	Baseline	End^#^		Baseline	End^#^	
**Fatigue**	6 (0)	14 (3)	< 0.001*	6 (0)	16 (5)	< 0.001*
**Pain characteristics**						
Pain Intensity	0 (0)	0 (5)	0.40	0 (0)	3 (5)	0.004*
Pain Area	0 (0)	0 (55.00)	0.06	0 (0)	4.00 (140.00)	0.009*
Pain Pressure Thresholds						
Masseter Muscles	100.00 (0)	95.50 (19.00)	0.75	100.00 (0)	97.00 (28.50)	0.56
Temporal Muscles	100.00 (0)	95.50 (23.00)	0.99	100.00 (0)	91.50 (21.50)	0.23
Index Finger (Reference)	100.00 (0)	91.00 (29.50)	0.98	100.00 (0)	102.50 (40.00)	0.24
**Functional Measures**						
Max Bite Force	100.00 (0)	104.60 (39.60)	0.30	100.00 (0)	96.70 (49.90)	0.12
Max Mouth Opening	100.00 (0)	98.20 (6.30)	0.25	100.00 (0)	100.00 (8.30)	0.56

The subjective fatigue was assessed in Borg’s RPE, the pain intensity (=peak pain) in numeric rating scale (NRS), the pain area in arbitrary units (au), the change in pressure pain threshold in percent (%), the change in maximum voluntary bite force in percent (%) and the change in maximum voluntary mouth opening capacity in percent (%). Data are expressed as median (IQR; interquartile range). P-values refer to the comparisons to baseline data by Friedman’s analysis of variance for repeated measures with Tukey post-hoc test. * = significant difference *P* < 0.05. #End refers to end of chewing

**Table 4:** Differences at end of chewing in all measures between trials and between 15 men and 16 women

	All		P-value	40-min		P-value	60-min		P-value
	40-min	60-min		Men	Women		Men	Women	
**Fatigue**	14 (3)	16 (5)	0.10	13 (7)	14.5 (3)	0.49	17 (4)	12.5 (6)	0.08
**Pain Characteristics**									
Pain Intensity	0 (5)	3 (5)	0.39	0 (3.5)	0 (5)	0.82	3 (6)	0 (3)	0.15
Pain Area	0 (55.00)	4.00 (140.00)	0.008*	0 (13.75)	5.00 (78.75)	0.12	2.00 (301.30)	67.00 (125.00)	0.91
Pressure Pain Thresholds									
PPT Masseter	95.50 (19.00)	97.00 (28.50)	0.71	99.25 (24.50)	85.50 (21.38)	0.02	95.25 (42.88)	97.50 (22.38)	0.43
PPT Temporal	95.50 (23.00)	91.50 (21.50)	0.57	100.50 (21.00)	95.50 (25.75)	0.45	86.00 (20.50)	95.50 (31.88)	0.20
PPT Reference	91.00 (29.50)	102.50 (40.00)	0.14	96.00 (29.00)	89.25 (32.50)	0.20	100.00 (45.50)	112.50 (28.13)	0.29
**Functional Measures**									
Max Bite Force	104.60 (39.60)	96.70 (49.90)	0.51	118.20 (49.60)	91.00 (53.40)	0.02	96.70 (36.10)	99.20 (85.70)	0.21
Max Mouth Opening	98.20 (6.30)	100.00 (8.30)	0.75	100.00 (6.20)	96.40 (5.90)	0.06	97.30 (8.30)	100.00 (4.90)	0.47

The subjective fatigue was assessed in Borg’s RPE, the pain intensity (=peak pain) in numeric rating scale (NRS), the pain area in arbitrary units (au), the change in pressure pain threshold in percent (%), the change in maximum voluntary bite force in percent (%) and the change in maximum mouth opening capacity in percent (%). Data are expressed as median (IQR; interquartile range). P-values refer to the comparisons between trials by Wilcoxon Signed Rank and sexes by Mann-Whitney Rank Sum test. * = significant difference P < 0.005 for subjective fatigue and pain intensity, < 0.013 for pain area and < 0.006 for the rest of the variables after applied Bonferroni Corrections

### Pain characteristics

The 60-min task induced a significant increase in pain intensity (=peak pain) and pain area (Table 3), while the 40-min task did not. Nonetheless, this significant increase did not even last to the first 10-min follow-up (Fig. 3b). There were no significant changes in PPT over the masseter, temporal muscles or in the index finger (reference point) when compared to baseline values, neither in the 40-min nor in the 60-min trials (Table 3). The induced pain area was significantly bigger in the 60-min trial compared to the 40-min trial (Table 4). There were no significant differences in pain intensity or change of PPT over the masseter, temporal muscles or the index finger when the two trials were compared.

### Functional measures

There were no significant changes regarding MVBF or maximum voluntary MOC after the 40-min chewing task or after the 60-min chewing task (Table 3), neither when the two chewing trials were compared (Table 4).

Differences between the sessions regarding subjective fatigue, pain characteristics and functional measures are presented in Table 5.

**Table 5:** Differences at end of chewing in all measures between the first and the second session based on data from 31 participants

	40-min Trial		P-value	60-min Trial		P-value
	First Session	Second Session		First Session	Second Session	
**Fatigue**	15 (3)	13 (6)	0.12	17 (4)	13 (9.50)	0.05
**Pain characteristics**						
Pain Intensity	0 (3.5)	0 (5)	0.69	3 (5)	1 (4)	0.74
Pain Area	36.00 (87.00)	0 (0)	0.002*	70.50 (139.25)	0 (66.00)	0.13
Pain Pressure Thresholds						
Masseter Muscles	97.50 (16.50)	94.50 (25.88)	0.83	98.50 (37.13)	97.00 (35.50)	0.51
Temporal Muscles	94.50 (13.00)	103.00 (31.75)	0.19	88.75 (22.88)	93.00 (32.00)	0.34
Index Finger (Reference)	91.50 (32.00)	88.25 (30.13)	0.30	102.00 (50.13)	107.50 (35.50)	0.96
**Functional Measures**						
Max Bite Force	97.10 (56.70)	105.30 (43.30)	0.20	76.10 (69.90)	99.90 (43.00)	0.12
Max Mouth Opening	96.60 (6.60)	100.00 (5.30)	0.13	96.10 (8.30)	100.00 (17.80)	0.13

The subjective fatigue was assessed in Borg’s RPE, the pain intensity (=peak pain) in numeric rating scale (NRS), the pain area in arbitrary units (au), the change in pressure pain threshold in percent (%), the change in maximum voluntary bite force in percent (%) and the change in maximum mouth opening capacity in percent (%). Data are expressed as median (IQR; interquartile range). P-values refer to the comparisons between sessions by Mann-Whitney Rank Sum test. * = significant difference P < 0.005 for subjective fatigue and pain intensity, < 0.013 for pain area and < 0.006 for the rest of the variables after applied Bonferroni Corrections

### Diagnoses according to diagnostic criteria of Temporomandibular disorders (DC/TMD)

All the participants were healthy pain-free individuals at study start. Thus, no DC/TMD diagnoses can be made and the diagnoses presented below can therefore be considered either as acute DC/TMD- or DC/TMD-alike diagnoses.

#### Myalgia

At the end of the 60-min chewing task 55% of the participants fulfilled the criteria for a diagnosis of myalgia, while only 39% of the participants fulfilled the criteria at the end of the 40-min chewing task. At the 2-h follow-up the number dropped to 26% of the participants for the 60-min chewing task and 23% for the 40-min chewing task. Only one participant fulfilled the criteria for a diagnosis of myofascial pain with referred pain at the end of the 60-min task, however this did not last to the 1-h follow-up.

#### Arthralgia

At the end of the 60-min chewing task 32% of the participants fulfilled the criteria for a diagnosis of arthralgia of the right temporomandibular joint (TMJ), 26% of the left and 23% bilaterally, while the corresponding amount of participants at the end of the 40-min chewing task were 29% for the right TMJ, 16% for the left and 13% bilaterally. At the 2-h follow-up of the 60-min chewing task this decreased to 10 and 13% for the right and left TMJ respectively and to 13% for both the right and left TMJs for the 40-min chewing task. Ten and 3% of the participants fulfilled the criteria of only arthralgia (without myalgia), all by palpation around the lateral pole of the joint, at the end of 40-min and 60-min chewing tasks respectively, however 32% of the participants displayed both myalgia and arthralgia at the end of 60-min chewing task, and 23% at the end of 40-min chewing task.

### Sex differences

#### Subjective fatigue

In men, there was a significant increase in subjective fatigue both in the 40-min and the 60-min trials when compared to baseline values (Table 6). This increase lasted for 20 min after the 60-min chewing task, while the increase did not last to the first 10-min follow-up in the 40-min trial. In women, there was a significant increase in fatigue in both the 40-min and the 60-min trials when compared to baseline (Table 7). In concordance to the men, this significant increase lasted for 20 min after the 40-min chewing task, while in the 60-min trial it did not last to the 10-min follow-up. When the sexes were compared, no significant differences were found between men and women in any of the trials (Table 4).

**Table 6:** Changes compared to baseline in all measures in 40- and 60- min trials in 15 men

	40-min Trial		P-value	60-min Trial		P-value
	Baseline	End^#^		Baseline	End^#^	
**Fatigue**	6 (0)	13 (7)	< 0.001*	6 (0)	17 (4)	< 0.001*
**Pain characteristics**						
Pain Intensity	0 (0)	0 (3.5)	0.85	0 (0)	3 (6)	0.04*
Pain Area	0 (0)	0 (13.75)	0.15	0 (0)	2.00 (301.30)	0.12
Pain Pressure Thresholds						
Masseter Muscles	100.00 (0)	99.25 (24.50)	0.17	100.00 (0)	95.25 (42.88)	0.21
Temporal Muscles	100.00 (0)	100.50 (21.00)	1.00	100.00 (0)	86.00 (20.50)	0.15
Index Finger (Reference)	100.00 (0)	96.00 (29.00)	0.22	100.00 (0)	100.00 (45.50)	0.28
**Functional Measures**						
Max Bite Force	100.00 (0)	118.20 (49.60)	0.09	100.00 (0)	96.70 (36.10)	0.58
Max Mouth Opening	100.00 (0)	100.00 (6.20)	0.45	100.00 (0)	97.30 (8.30)	0.23

The subjective fatigue was assessed in Borg’s RPE, the pain intensity (=peak pain) in numeric rating scale (NRS), the pain area in arbitrary units (au), the change in pressure pain threshold in percent (%), the change in maximum voluntary bite force in percent (%) and the change in maximum voluntary mouth opening capacity in percent (%). Data are expressed as median (IQR; interquartile range). P-values refer to the comparisons to baseline data by Friedman’s analysis of variance for repeated measures with Tukey post-hoc test. * = significant difference P < 0.05. #End refers to end of chewing

**Table 7:** Changes compared to baseline in all measures in 40- and 60- min trials in 16 women

	40-min Trial		P-value	60-min Trial		P-value
	Baseline	End^#^		Baseline	End^#^	
**Fatigue**	6 (0)	14.5 (3)	< 0.001*	6 (0)	12.5 (6)	0.005*
**Pain characteristics**						
Pain Intensity	0 (0)	0 (5)	0.86	0 (0)	0 (3)	0.58
Pain Area	0 (0)	5.00 (78.75)	0.05	0 (0)	67.00 (125.00)	0.08
Pain Pressure Thresholds						
Masseter Muscles	100.00 (0)	85.50 (21.38)	0.19	100.00 (0)	97.50 (22.38)	0.77
Temporal Muscles	100.00 (0)	95.50 (25.75)	0.95	100.00 (0)	95.50 (31.88)	0.91
Index Finger (Reference)	100.00 (0)	89.25 (32.50)	0.87	100.00 (0)	112.50 (28.13)	0.50
**Functional Measures**						
Max Bite Force	100.00 (0)	91.00 (53.40)	0.17	100.00 (0)	99.20 (85.70)	0.82
Max Mouth Opening	100.00 (0)	96.40 (5.90)	0.13	100.00 (0)	100.00 (4.90)	0.17

The subjective fatigue was assessed in Borg’s RPE, the pain intensity (=peak pain) in numeric rating scale (NRS), the pain area in arbitrary units (au), the change in pressure pain threshold in percent (%), the change in maximum voluntary bite force in percent (%) and the change in maximum voluntary mouth opening capacity in percent (%). Data are expressed as median (IQR; interquartile range). P-values refer to the comparisons to baseline data by Friedman’s analysis of variance for repeated measures with Tukey post-hoc test. * = significant difference P < 0.05. #End refers to end of chewing

#### Pain characteristics

In men, there was a significant increase in pain intensity in the 60-min trial when compared to baseline, which was not found in the 40-min trial (Table 6). The significant increase did not last to the 10-min follow-up. On the other hand, in women no significant changes were found compared to baseline neither in the 40-min nor in the 60-min trial (Table 7). Further, the pain area in men did not change compared to baseline values in the 40-min trial but tended to increase, although not significantly, in the 60-min trial (Table 6). The pain area tended to increase in women but the increase was not significant in the 40-min and 60-min trials (Table 7). There were no changes in PPT in any of the assessment points when compared to baseline, neither in men nor in women in any of the trials (Tables 6 and 7). No significant differences were found in pain characteristics between the sexes neither in the 40-min nor in the 60-min trial (Table 4).

#### Functional measures

Maximum voluntary bite force and maximum voluntary MOC showed no significant changes within the two sex groups compared to baseline (Tables 6 and 7). No significant differences were found between the sexes in any of the trials (Table 4).

## Discussion

The main finding of the study was that 40-min of chewing hard gums only induced high levels of subjective fatigue, while 60-min of chewing also induced mild levels of pain and a larger area of pain spread. These findings are in agreement with previous studies where excessive chewing evoked fatigue [11, 12, 30] and pain [11, 12] in human jaw. The levels of perceived pain and subjective fatigue were comparable with TMD-related pain reported in earlier studies [6, 7]. However, the results also indicate that the chewing task duration needs to be longer in order to induce significant pain intensity that may be clinically relevant [31] especially in women and with pain duration that allows further investigations. Correlation analysis made in a previous study [32] pointed towards a stronger association between measures of electromyographic muscle activity (EMG) and fatigue rather than low intensive pain which thus explains the more obvious increase in fatigue seen in our study. Probably pain may be induced and intensified as a protective mechanism after a prolonged jaw activity or when chewing on harder food. It is suggested that human jaw-closing muscles contain more fatigue-resistant slow fibers (type I) than fatigue-resistant (type IIA) or fatigue susceptible fast fibers (type IIB) [33]. In this study the time of the chewing tasks was pre-determined and might not be enough to reach the levels of fatigue where all fatigue-susceptible fast fibers (type IIB) are recruited [34]. This could be a possible explanation to the mild pain intensity and the fast immediate recovery after the chewing task.

Since one of the objectives was to investigate if the induced pain and fatigue could have a duration that would allow immediate further investigations of the jaw function, no recordings were assessed after 24 h and 48 h in our study. Previous studies using chewing gum as a pain-inducing model did not show any delayed increase in fatigue or pain intensity scores, i.e. delayed onset muscle soreness (DOMS) [11, 12]. Muscle hyperactivity causes ischemia in the muscles and accumulation of metabolic products such as potassium, adenosine and lactate, which explain the induced subjective fatigue and pain after the excessive chewing [10, 32]. Since chewing gum as hard as the one used in our study can induce up to 50% of maximum EMG activity [12] ischemia might occur [35]. The hyperactivity causes an excitation of group III and IV muscle afferents [36] but also a reflex inhibition of the motor-neurons as a protective mechanism later on [37, 38]. The quick muscle recovery shortly after the end of the chewing due to restored blood flow through the high density of capillaries [39] in jaw muscles driving the metabolic accumulations away and re-oxygenating these muscles may be explained by the fact that the participants were healthy individuals [12].

In accordance with previous studies, excessive chewing did not induce, any significant effects on PPTs [12], BF [40] or MOC. The induced pain was a mild localized (non-referred) pain which might explain the non-significant effects [29]. However, these findings are in contrast to other studies [11, 32, 41, 42] showing an inconsistency in results from previous studies. An explanation for such an inconsistency may be the different studies’ populations. Also, the pain-inducing/fatiguing tasks used in many of those studies were clenching or stretching not chewing. Furthermore, those studies included different task-durations and time-points when the assessments were recorded.

The non-significant decrease of the MVBF in the 60-min trial might be explained by an activity compensation in other non-affected muscle parts or muscles [43–49] or a modification in sensory input from muscle receptors that were affected by the increased intercuspal distance that led to stretching the fibers in the jaw-closing muscles [50]. Since our study included assessing bite forces, a transducer had to be placed in-between the teeth. In our study, the maximum voluntary MOC in the 60-min trial did not show any significant change which was contradicted to previous studies. Those studies showed that longer period of excessive chewing caused rigidity of the masseter muscles [51], also that the experimental muscle pain facilitated the gamma motor neurons suggesting an increased reflex stiffness in the muscles [52]. A proposed strategy for what happened in our trial might be changes in the motor activity in order to allow the non-affected parts of the muscle or other muscles to continue to work as normal, which would enable optimal muscle function even during pain [44–49]. Also, the trial might affect the closing jaw muscles in a bigger extension than the opening jaw muscles.

The majority of participants displayed myalgia-like diagnosis in both chewing tasks but also arthralgia-like diagnosis. Those decreased by time but there were still painful TMD-like diagnoses that would be fulfilled at 2-h follow-up which did not seem to be in accordance with the reported pain intensity (at rest). The provocation (especially palpation) used in the clinical examination for diagnosing TMD might be the reason of this discrepancy between the findings. Only one participant showed myofacial pain with referred pain indicating that the chewing task was probably not strong enough as a noxious stimulus [53]. The arthralgia-like diagnosis occurred in higher percent in the right TMJ. Further analysis showed that the habitual/preferred chewing side was the right side for the majority of the participants. This is in line with results from the previous studies that demonstrated that the habitual chewing side had a significantly higher EMG activity, indicating that the load on the habitual side is higher [30, 54].

### Sex differences

Subjective fatigue increased significantly compared to baseline values in both men and women in 40-min and 60-min trials. No significant sex differences were found even if the fatigue-resistant slow type I fibers in masseter muscles have a significantly larger diameter in women than men, while for the type II fibers it is the other way around [55]. Type I motor units are recruited first [56] and muscle fibers with larger diameters produce faster action potentials [57]. The results seem to suggest that women recovered faster from the induced fatigue compared to men in the 60-min trial, which is probably be related to men expressing pain after the 60-min chewing task.

An interesting finding was that in men pain intensity increased significantly compared to baseline values in the 60-min trial. Still, no significant sex differences were found regarding the levels of pain intensity or pain area in both 40-min and 60-min trials. This is in line with results from a previous study [42] that also showed no sex differences in pain intensity and area. However, these results are contradictory to those from a previous study where women reported a higher level of pain intensity as well as a larger pain area [58]. Perhaps the sensation of subjective fatigue exceeded the sensation of pain and thereby affecting the men’s subjective assessment of pain. No sex differences could be shown regarding the changes in PPTs in our study. This is in line with previous studies where PPTs remained unchanged in women [12] and in men [32] after excessive chewing or clenching. However, there is an inconsistency in results from previous studies since there were other studies showing that women had lower PPT values than men [58] or a significant decrease in PPT until 24 h after a 100 min chewing task in men [11]. An explanation to the discrepancies could be the different methodologies. Moreover, sex differences are more obvious in longer-lasting pain conditions [59].

### Strength and limitations

The clinical examination was performed by one examiner only who was blinded to the duration of the trial and used the standardized and robust protocol DC/TMD [4]. The study was divided into two sessions with a wash-out period of 1 week in order to avoid any contamination of the data due to DOMS [8, 9, 60]. The included men and women were age matched in order to analyze any possible sex differences [58]. There were further no differences in any of the bio-psycho-social variables between the participants who started with 40-min or 60-min chewing, which confirmed the counterbalancing and enhanced the homogeneity of the population recruited.

One possible limitation could be the fact that we did not have enough material to account for the menstrual cycle. The phases of menstrual cycle were asked for but not taken into consideration since as all participating women were in different menstrual phases. Hence this study could be seen as representative for the population and it had been showed that intra-individual variability in the pain response is greater than the influence of estrogen [61]. The self-reported bruxism could be considered an information bias since nightly bruxism could still occur without the individual’s knowledge. The examiner and the participants could count the amount of chewing bouts to find out which was the longer duration trial, and thus not being completely blinded. During-chewing-data were assessed, however, in order to avoid any confusion between during task and follow-up time-points we chose not to present those data. Women seemed to recover faster form the induced fatigue after the 60-min trial compared to the 40-min trial which might appear puzzling. A possible selection bias that might occur due to women dropping-out from the second session at which they were randomized to the 60-min chewing trial could explain the finding and would be considered a drawback.

## Conclusions

Based on the findings of this and previous studies excessive chewing in its current form does not seem to be a proper pain experimental model. The model needs further adjustments in order to mimic TMD-pain especially in women, to induce referred pain and to prolong the pain duration.

### Future studies

- The excessive chewing model can be used in future studies as an experimental fatigue model.
- The excessive chewing model needs further modification if it is aimed to be used as a pain model in future studies.
- In order to mimic the clinical fatigue and pain in TMD patients, combining the excessive chewing model with an algesic injection may provide a proper experimental model in future studies.

## Data Availability

Data used in this trial can be provided by the corresponded author on a reasonable request.

## Abbreviations

ANOVA: Analysis of variance
au: Arbitrary units
MVBF: Maximum voluntary bite force
Borg’s RPE: Borg’s rating of perceived exertion
DC/TMD: Diagnostic criteria for temporomandibular disorders
DOMS: Delayed onset muscle soreness
EMG: Electromyographic muscle activity
GAD-7: Generalized anxiety disorder scale-7
IQR: Interquartile range
kPa: Kilo Pascal
Max: Maximum
MOC: Mouth opening capacity
NRS: Numeric rating scale
PCS-13: Pain catastrophizing scale-13
PHQ-15: The patient health questionnaire for physical symptoms-15
PHQ-9: The patient health questionnaire for depression-9
PPT: Pressure pain threshold
PSS-10: Perceived stress scale-10
SCON: Scandinavian Center for Orofacial Neurosciences
SD: Standard deviation
TMD: Temporomandibular disorders
TMJ: Temporomandibular joint
